# Relative Abundance and Plasmodium Infection Rates of Malaria Vectors in and around Jabalpur, a Malaria Endemic Region in Madhya Pradesh State, Central India

**DOI:** 10.1371/journal.pone.0126932

**Published:** 2015-05-13

**Authors:** Neeru Singh, Ashok K. Mishra, Sunil K. Chand, Praveen K. Bharti, Mrigendra P. Singh, Nutan Nanda, Om P. Singh, Kranti Sodagiri, Venkatachalam Udhyakumar

**Affiliations:** 1 National Institute for Research in Tribal Health (formerly known as Regional Medical Research Centre for Tribals), Nagpur Road, Garha, Jabalpur, Madhya Pradesh, India; 2 National Institute of Malaria Research, ICMR, Field Station, Jabalpur, Madhya Pradesh, India; 3 National Institute of Malaria Research, New Delhi, India; 4 Genetics and Immunology Laboratory, Malaria Branch, DPD, NCZVED, CCID, CDC, Atlanta United States of America; University College London, UNITED KINGDOM

## Abstract

**Background:**

This study was undertaken in two Primary Health Centers (PHCs) of malaria endemic district Jabalpur in Madhya Pradesh (Central India).

**Methods:**

In this study we had investigated the relative frequencies of the different anopheline species collected within the study areas by using indoor resting catches, CDC light trap and human landing methods. Sibling species of malaria vectors were identified by cytogenetic and molecular techniques. The role of each vector and its sibling species in the transmission of the different *Plasmodium* species was ascertained by using sporozoite ELISA.

**Results:**

A total of 52,857 specimens comprising of 17 anopheline species were collected by three different methods (39,964 by indoor resting collections, 1059 by human landing and 11,834 by CDC light trap). *Anopheles culicifacies* was most predominant species in all collections (55, 71 and 32% in indoor resting, human landing and light trap collections respectively) followed by *An*. *subpictus* and *An*. *annularis*. All five sibling species of *An*. *culicifacies* viz. species A, B, C, D and E were found while only species T and S of *An*. *fluviatilis* were collected. The overall sporozoite rate in *An*. *culicifacies* and *An*. *fluviatilis *were 0.42% (0.25% for *P*. *falciparum* and 0.17% for *P*. *vivax*) and 0.90% (0.45% for *P*. *falciparum* and 0.45% for *P*. *vivax*) respectively. *An*. *culicifacies* and *An*. *fluviatilis* were found harbouring both *P*. *vivax* variants VK-210 and VK-247, and *P*. *falciparum*. *An*. *culicifacies* sibling species C and D were incriminated as vectors during most part of the year while sibling species T of *An*. *fluviatilis* was identified as potential vector in monsoon and post monsoon season.

**Conclusions:**

*An*. *culicifacies* species C (59%) was the most abundant species followed by *An*. *culicifacies* D (24%), B (8.7%), E (6.7%) and A (1.5%). Among *An*. *fluviatilis* sibling species, species T was common (99%) and only few specimens of S were found. Our study provides crucial information on the prevalence of *An*. *culicifacies* and *An*. *fluviatilis* sibling species and their potential in malaria transmission which will assist in developing strategic control measures against these vectors.

## Introduction

Malaria is a global health problem. In India, malaria is highly endemic in Madhya Pradesh (MP). MP is the fourth highly malarious state in the country, contributing 7% of total malaria cases [1]. At present the malaria control programme is confronted by several technical (development of double/triple insecticide resistance, inadequate supplies of insecticide and poor spray coverage) and administrative difficulties (shortage of human resources, transport and funds etc) that reduce the effectiveness of the malaria control programme. The success of vector control programme relies on correct identification of malaria vectors. Morphotaxonomic identification of vectors is very difficult owing to overlapping morphological characteristics of closely related species [2]. Vector heterogeneity is extensive in MP where at least three anopheline species are implicated as potential vectors of malaria. Two vector species found commonly in the study area, are *Anopheles culicifacies* Giles and *An*. *fluviatilis* James [3], [4]. Both these vectors are species complexes and belong to subgenus Cellia and Series Myzomyia and together transmit about 80% of malaria in the country [5]. However, we know very little about the contribution of individual species to malaria prevalence in heterogeneous environments. Although some entomological studies were conducted earlier in MP [6], [7], only limited information is available in understanding the role of vectors with special reference to sibling species in malaria transmission [3], [8]. Moreover, there has been an ecological succession of vector species in different areas and there is a need to study the changing pattern of vector behavior [9]. Further, vector control measures applied in most of the state have been sporadic, using indoor residual spray (IRS) without the basic knowledge on the bionomics of vectors in the area [10].

Different studies have shown distinct differences among population of *An*. *culicifacies* and *An*. *fluviatilis* in feeding preference, resting behavior and infection rates in different states [11], [12], [13], [14]. These differences are likely to be due to the fact that these are a complex of sibling species with differing behaviours and vector capacities. Therefore, in this study, we investigated the relative frequencies of the different anopheline species collected within the study area according to the ecotypes, seasons and method of capture and the role of each vector and its sibling species in the transmission of different *Plasmodium* species. These results provide a better understanding of relative abundance and Plasmodium infection rates of malaria vectors in the study area and represent important baseline data essential to measure the impact of control strategies or launching new intervention measure.

## Materials and Methods

### Ethics Statement

This study was approved by the ethical research committee of National Institute of Malaria Research New Delhi, National Institute for Research in Tribal Health (formerly known as Regional Medical Research Center for Tribals), Jabalpur and Center for Disease Control and Prevention, CDC, Atlanta, GA, USA. No specific permission was required for conducting studies in villages of the Bargi Primary Health Centre (PHC) and Sihora PHC and this field study did not involve endangered or protected species.

### Study site

**Fig 1:**
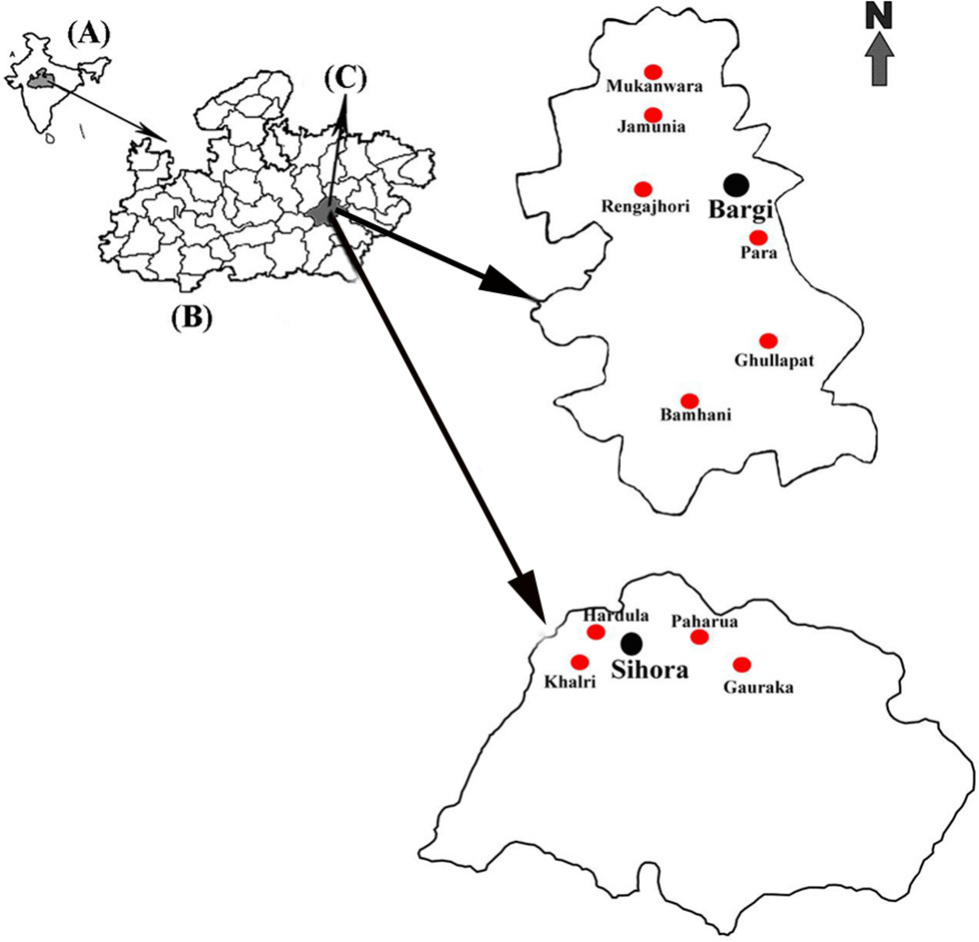
Map of India (A) showing Madhya Pradesh (B), Jabalpur district (C) and Bargi and Sihora Primary Health Centres.

This study was undertaken in Bargi and Sihora PHCs of Jabalpur district (Latitude 23° 16’ N; Longitude 79° 59’ E). The district is divided into 7 blocks and 22 PHCs of which 2 PHCs, Bargi—Site A (coordinates 22° 58’ 60 N—79° 52’ 0 E; 405 MSL) and Sihora—Site B (coordinates 23° 28’ 60 N—80° 70’ 0 E; 358 MSL) are selected for this study. Bargi PHC is about 30 km from Jabalpur while Sihora PHC is 40 km from Jabalpur in opposite directions (Fig 1). This study was undertaken from August 2006 to May 2010 in ten villages which are approachable throughout the year and represent various geographical ecotypes.

#### Bargi PHC (Site A)

Bargi PHC has a geographical area of 1634 sq km of which 364 sq km is under forest. In all 6 villages were selected from this PHC, of which 3 are foothill villages. These are Mukanwara, Jamuniya and Para and their total population is 2357 (general pop 0.98%, other backward caste (OBC) 39%, schedule caste (SC) 4.3% and schedule tribe (ST) 56%). Two villages are in forest i.e. Bamhni and Rengajhori with a total population of 2051 (gen pop 1.5%, OBC 10%, SC 2.1% and ST 86%) while one village is near dam reservoir i.e. Gullapath with a population of 565 (gen pop 1%, OBC 49%, SC 7% and ST 43%). These villages are divided into 4–10 hamlets and are 15–40 km from the main road and 5–15 km from each other. Several seasonal and perennial streams criss cross the villages providing innumerous breeding sites. The tropical deciduous forest of the area consists of mostly *Tectona grandis* (Teak), *Shorea robusta* (Sal), *Madhuca indica* (Mahua) and *Bambusa nutanus* (Bamboo). People are mostly less educated and less wealthy and work mainly as casual worker in forest nursery, road construction or as agriculture labourers. The literacy rate is 45% and economy is forest based.

Their houses are scattered in agricultural fields and in forest and are made of mud, thatch and bamboo. The dwellings are small with low doors, dark, humid, without ventilation and often have one point electricity connection. Very often cattles are also sheltered in the house. Drinking water is brought from wells or streams or hand pump. The public transport facilities are non-existent. Villages had not received IRS at the time of this study.

#### Sihora PHC (Site B)

The geographical area of Sihora PHC is 1097 sq km. The 4 villages undertaken in the study are in plain, thickly populated and the natives are relatively more prosperous. These villages are Gauraha, Paharua, Khalari, Hardua with a total population of 9217 (gen pop 6.7%, OBC 47%, SC 9.8% and ST 36.7%). These villages are more compact and divided into 4–5 hamlets. Residents seldom work away from the villages and the local economy is agriculture based. Houses are more spacious, better ventilated, have electricity and are made of bricks and cement. The medical facilities are much better and villages are having piped water supply or use hand pump. These villages are having metal road with better transport facilities. The literacy rate is 53%. The IRS was not carried out during the period of this study.

The climate is similar at both the sites and is characterized by a hot summer (April-June), a monsoon/rainy season (July-September), a post monsoon (October-November), a cool autumn season (December-January) and a spring season (February-March). The mean annual rainfall for district Jabalpur is 1400mm. The mean maximum temperature ranges between 20.5 and 44°C with May being the hottest month and the mean minimum temperature ranges between 3.5 and 25°C with December being the coldest month.

### Entomological monitoring

Indoor resting mosquitoes were collected once in a month from 10 villages (6 villages in Bargi, site A and 4 villages in Sihora, site B). Anophelines resting inside 4 fixed houses (same structures were sampled each time) and 4 randomly selected houses located in different parts of the villages (4 human dwellings and 4 cattle sheds were sampled during early morning (0600h - 0800h) for 15 minutes each place following standard protocol [15]. A team of 4 insect collectors was assigned to each village. The same collectors caught mosquitoes with a flashlight and mouth aspirators in each study village.

Human landing catches [HLC, indoor (I) & outdoor (O)] were made from 1800 to 0600h in the same villages of site A and site B. Catches were made by 4 collectors (441 man night indoor and 392 man night outdoor, outdoor collections were not made during extreme winter and during excessive rains) equipped with flashlights and aspirators seated facing each other on stools on verandahs where people sleep during the night [16]. Catches on verandahs were considered as indoor catches and a 2^nd^ pair of collectors similarly equipped was seated outdoors about 5 meters away from the houses. The feet, legs, arms and heads of the collectors were bare. Indoor and outdoor teams exchanged positions hourly to avoid collector bias. During the catches, a trained Research Assistant_._ identified the mosquitoes morphologically and stored in a paper cup labeled by site and unique village identification code.

CDC light trap (LT) was used in the same villages from site A and B. The traps were run for a period of 220 nights from 1800 to 0600h (September, 2006—May, 2010). Traps were always fixed at a constant height of 5.5 feet at fixed locations outdoors near occupied human dwellings and indoors in human dwellings in each village. Each trap was emptied manually at hourly intervals until morning [17].

### Laboratory processing of anophelines

#### Sibling Species of Anopheles culicifacies by cytogenetic method

Mosquitoes were morphologically identified in the laboratory using standard keys [18], [19]. Sibling species of *An*. *culicifacies* were identified by cytogenetic method. Ovaries of *An*. *culicifacies* from half-gravid females were pulled and preserved in modified Carnoy's fixative (1 part acetic acid, 3 parts methanol). Ovaries were processed in 50% propionic acid and stained with 2% lacto-aceto-orcein according to the method of Green and Hunt [20] for the preparation of polytene chromosome. Sibling species of *An*. *culicifacies* were identified following standard protocol [21].

### Molecular methods of sibling species identification

#### DNA Isolation and PCR amplification

Genomic DNA was isolated from both head–thoracic region and abdominal region of individual mosquitoes separately by phenol–chloroform method [22] and re-suspended in 200μL of TE buffer (10mM Tris-HCl, 1mM EDTA, pH 8.0) for further experimentation. Identification of sibling species of *An*. *fluviatilis* was done using 28S-rDNA based PCR assay developed by Singh et al [23]. Identification of sibling species of *An*. *culicifacies* was done by using the primer for D3 region of 28S rDNA [24] and ITS2 region [25]. In brief the PCR using universal primers 20 pmol, D3A (5-GAC CCG TCT TGA AAC ACG GA-3) and D3B (5-TCG GAA GGA ACC AGC TAC TA-3), 200 μM of each dNTP, 1.5 mM MgCl_2_, 1x PCR buffer and 1 unit of taq DNA polymerase. The cycling condition was initial denaturation at 95°C for 5 min followed by 40 cycles each of denaturation at 95°C for 40 s, annealing at 48°C for 45 s and extension at 72°C for 1 min, followed by a final extension at 72°C for 7 min.

### DNA-sequencing

The PCR product of D3 and ITS2 gene was excised from the gel, purified, and sequenced from both directions using BigDye Terminator Ready Reaction Kit. For each sequencing reaction, 50 ng of purified PCR product was mixed with a reaction mixture containing 2.5x sequencing buffer and big dye terminator (Applied Biosystems, Foster City, CA). Cycle sequencing parameters used were as follows: 96°C for 1 min followed by 25 cycles of 96°C for 10 s, 50°C for 5 s and 60°C for 4 min. The PCR product was purified. Sequences were analyzed on the ABI Prism 3130 XL Genetic Analyzer (Applied Biosystems). Alignment of sequences was done with using DNASTAR software (DNASTAR, Inc., Madison, WI). The sequences generated were submitted to GeneBank.

### Processing for vector incrimination

Head and thorax of individual *An*. *fluviatilis* and *An*. *culicifacies* were processed for sporozoite detection in the salivary glands by sporozoite-ELISA [26], [27] which detects circumsporozoite (CS) proteins using *P*. *vivax* (210 & 247) and *P*. *falciparum* specific monoclonal antibodies. Briefly aliquots (50μL) of the capture CS-antibody (2μg/ml PBS) were pipetted into the wells of flexible polyvinyl chloride (PVC) U-shaped, 96 well microtitre plates and stored overnight at 4°C. The plate was blocked with blocking buffer (PBS Casein) for 1 hour. Samples were prepared in blocking buffer and added to the wells (duplicate), incubated for 2 hours and washed with washing buffer (PBS with 0.1% Tween-20). 50 μL of each peroxidase-conjugated monoclonal antibody (2μg/ml blocking buffer) was added to each well and incubated for 1 hour. The contents of the wells were subsequently aspirated, the wells washed three times with PBS-0.05% Tween 20 (PBS-T), and 100μL of Tetramethylbenzidine substrate (TMB) was added to each well. The absorbance of reactions was determined at 450 nm using a microplate reader.

### Sample Size estimation

#### Sibling species

The preliminary studies carried out in 1988 on sibling species of *An*. *culicifacies* in Jabalpur, MP showed that species C is the most predominant species (65–90%) and the relative proportion of other species varies, i.e. 1%-15% [3]. However, no new study is available on sibling species from this area and the sibling species distribution is highly seasonal. The species A is rare, hence assuming the prevalence of species A as 3%, the sample size was estimated at 5% level of significance and 50% relative precision, i.e. the estimated species A may vary from 1.5%-4.5%. We need a minimum sample of 517 *An*. *culicifacies* for sibling species study. A sample of 517 will be also enough for all other species.

#### Sporozoite rate among vectors

Recent studies carried out in this part of MP show that the sporozoite rate among mosquitoes varies from 0.4%- 0.8% [8], [10]. Thus assuming a sporozoite rate as 0.5%, the sample size was estimated at 5% level of significance and 50% relative precision, i.e. the estimated sporozoite rate may vary from 0.25%-0.75%. We need a minimum sample of 3184 *An*. *culicifacies* mosquitoes for testing. For *An*. *fluviatilis*, since the number was small hence all available specimens were tested.

### Data analysis

Categorical data are presented as frequency counts (percent) and compared using the Pearson’s chi-square test. Continuous data are summarized as mean (± standard deviation and compared using one way analysis of variance (ANOVA) with Bonferroni adjustment for multiple comparisons. Log transformed data was used in ANOVA. Statistical analysis was performed using STATA software version 8.0 (StataCorp, LP, TX, USA)).

## Results

### Man hour density (MHD)

**Fig 2:**
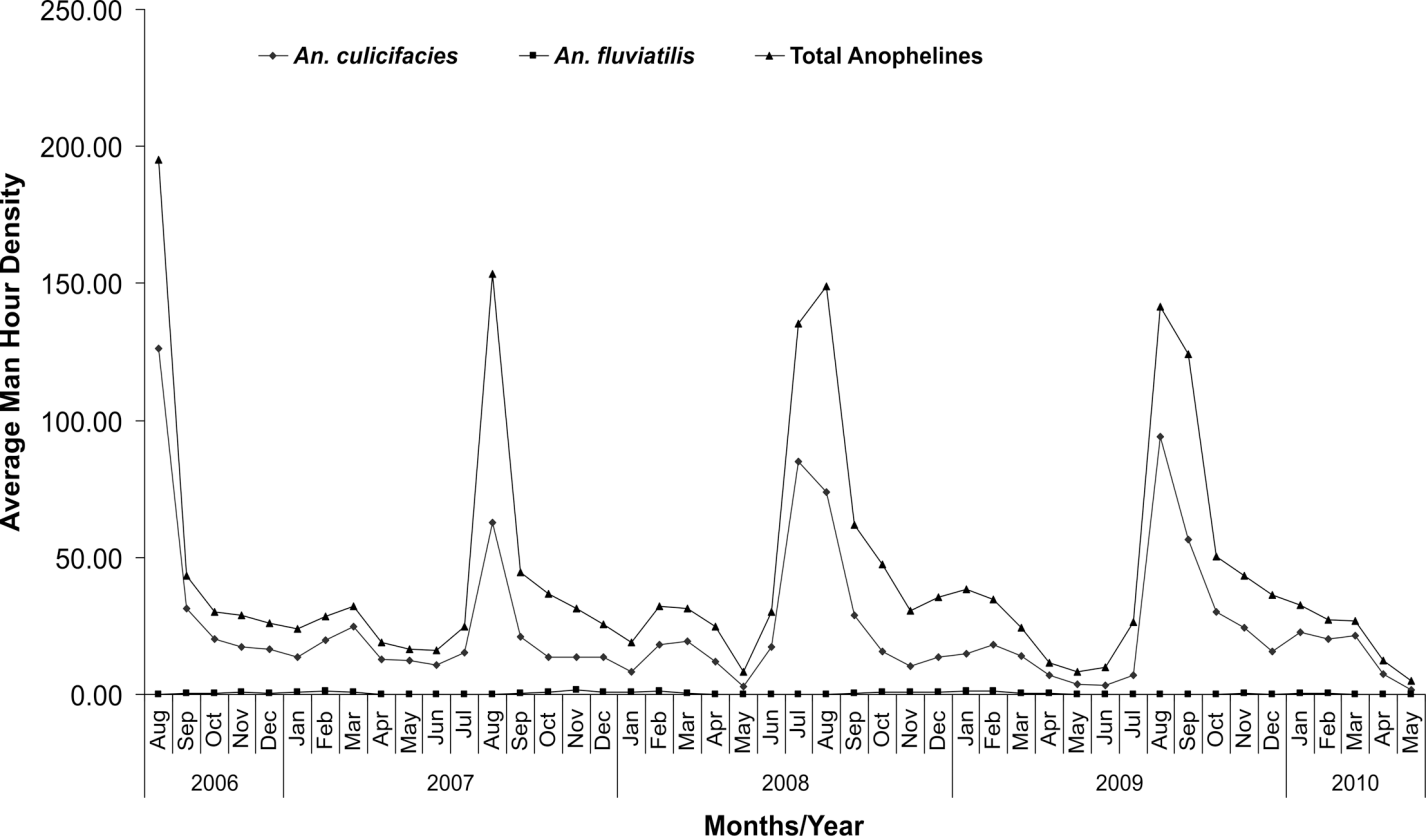
Month/Year wise man hour density (Indoor Resting Collections) in study villages of district Jabalpur.

The anopheline fauna of the study villages comprised of 16 species of which *An*. *culicifacies* Giles (54.8%), *An*. *subpictus* Grassi (28.7%), *An*. *annularis* Van Der Wulp (13.6%) were the most abundant species in indoor resting collections (Table 1). *An*. *fluviatilis* James, *An*. *vagus* Donitz, *An*. *pallidus* Theobald, *An*. *barbirostris* Van Der Wulp, *An*. *nigerrimus* Giles, *An*. *splendidus* Koidzumi, *An*. *theobaldi* Giles, *An*. *aconitus* Doenitz, *An*. *varuna* Iyengar, *An*. *maculatus* Theobald, *An*. *tessellatus* Theobald, *An*. *turkhudi* Liston, *An*. *pseudojamesi* Strickland and Chaudhary were collected in very small numbers. The number of anopheline mosquitoes collected throughout the year did not show a consistent pattern and month to month variations in the number of mosquitoes were common (Fig 2). The 894 man hours of efforts revealed that *An*. *culicifacies* is most predominant species followed by *An*. *subpictus*. The relative abundance of *An*. *culicifacies* was lowest in May-June (7.76 ± 13.65) and highest in August-September (61.80 ± 68.53).

**Table 1:** Number and proportion of anophelines caught by different methods in study villages of district Jabalpur.

Species	Indoor resting (%)	Human landing (%)	Light trap catches (%)
*An*. *culicifacies*	21920 (54.8%)	755 (71.3%)	3828 (32.3%)
*An*. *fluviatilis*	381 (0.95%)	17 (1.6%)	213 (1.8%)
*An*. *subpictus*	11481 (28.7%)	183 (17.3%)	3314 (28.0%)
*An*. *annularis*	5429 (13.6%)	61 (5.8%)	3327 (28.1%)
*An*. *vagus*	186 (0.46%)	7 (0.7%)	182 (1.54%)
*An*. *pallidus*	449 (1.12%)	16 (1.52%)	285 (2.42%)
*An*. *splendidus*	36 (0.09%)	1 (0.09%)	80 (0.7%)
*An*. *barbirostris*	30 (0.07%)	15 (1.43%)	150 (1.3%)
*An*. *theobaldi*	23 (0.06%)	0 (0.0%)	115 (1.0%)
Other *Anopheles*	29 (0.07%)	4 (0.4%)	340 (2.9%)
**Total**	**39964**	**1059**	**11834**

### Light trap (LT) catches

**Fig 3:**
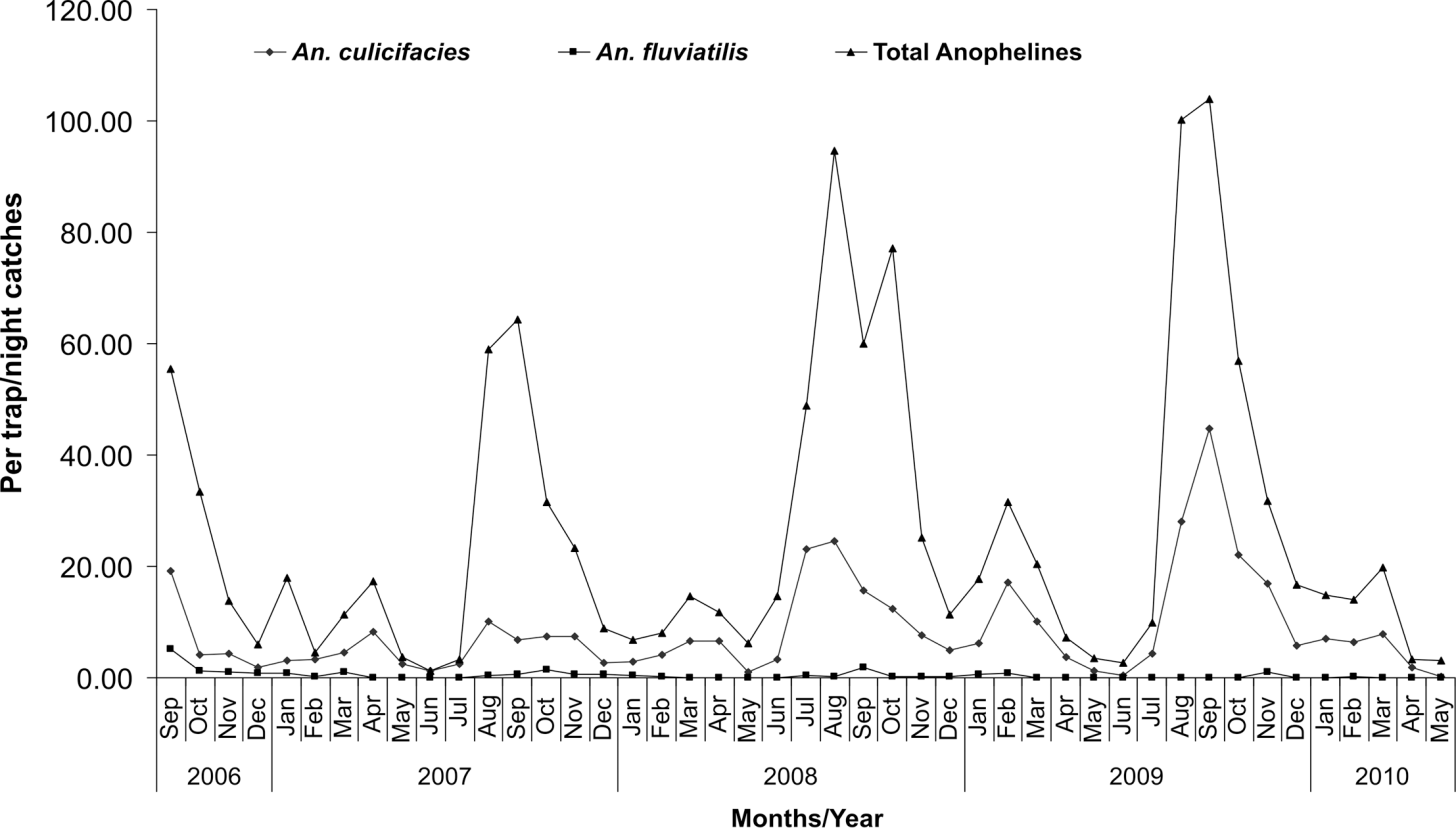
Month/Year wise per trap per night catches in study villages of district Jabalpur.

**Fig 4:**
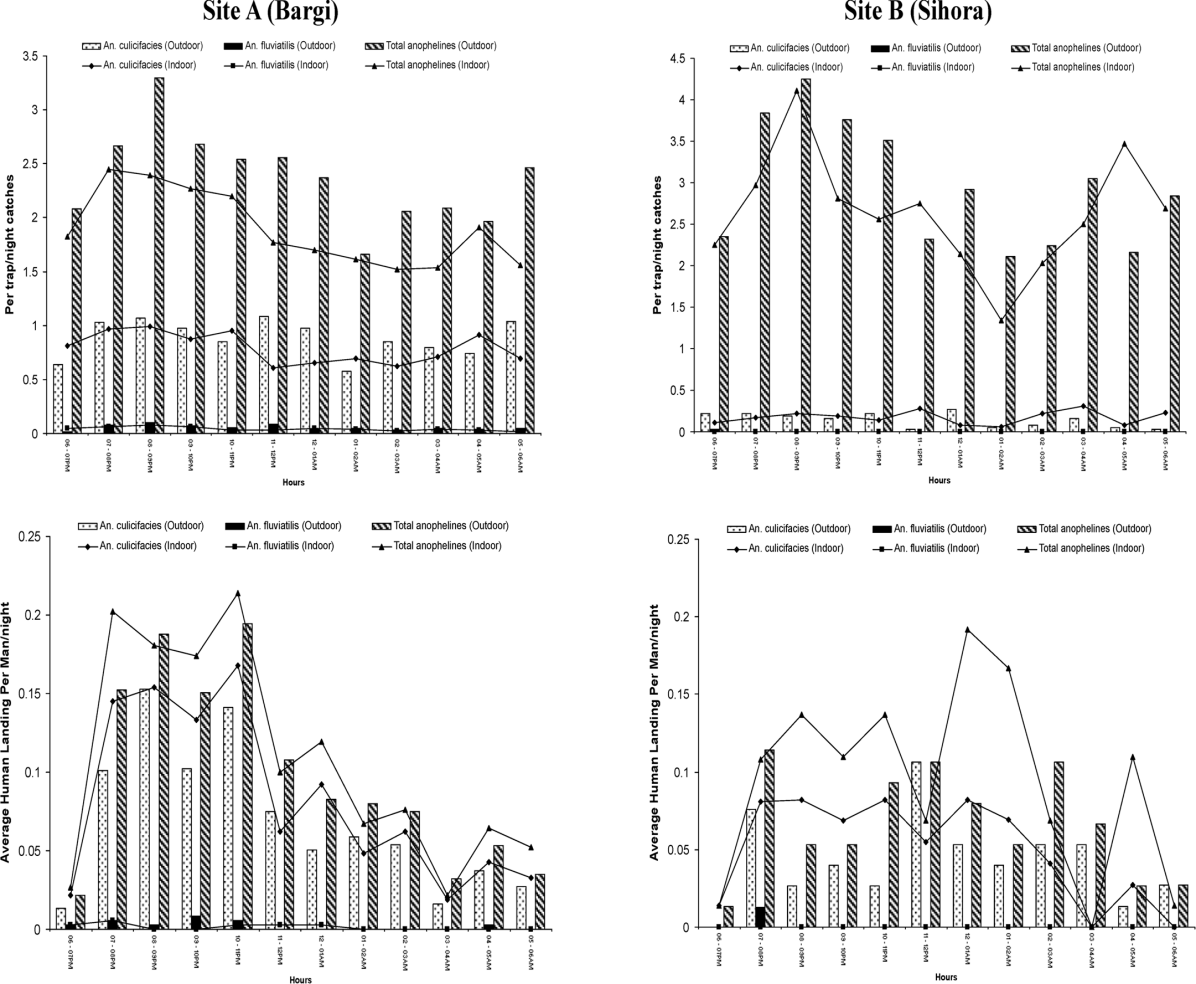
Showing hourly trap catches and human landing catches of *An*. *culicifacies* and *An*. *fluviatilis* in indoor and outdoor in study villages of district Jabalpur.

LT catches showed one more species (*An*. *jeyporiensis* James) in very small numbers in addition to 16 species found in MHD (Table 1). The per trap/night catches of *An*. *culicifacies* was observed 4.97±5.40, 9.39±11.11, 13.39±11.18 in the year 2007, 2008 and 2009 respectively and showed a significant increase in 2008 and 2009 when compared with 2007 (p<0.0001). A similar trend was observed in total anophelines which was 20.53±21.33, 31.61±24.26, 33.56±17.64 respectively in 2007, 2008 and 2009 and this is also significant statistically (p<0.0001) (Fig 3). However, *An*. *fluviatilis* showed a decreasing trend from 2007 onwards and it was not significant. Analysis by ecotype revealed that *An*. *culicifacies* was lowest in plain villages as compared to dam, forest and foothill villages and this difference was highly significant (p<0.0001). Highest catches of *An*. *fluviatilis* was recorded in dam village followed by foothill as compared to plain villages (p = 0.031). Hourly collections showed that mosquitoes were trapped almost in every hour of collection in both indoor and outdoor sites. Highest trap catches of anopheles were obtained between 1900–2300 hrs while highest trap catches of *An*. *culicifacies* was between 1900–2100 hrs both in light trap indoor (LTI) and light trap outdoor (LTO) collections (Fig 4). Further, analysis revealed that relatively higher per trap/night catches of total anophelines and of *An*. *culicifacies* were obtained in the month of August-September (77.8±58.18, 21.25±23.59) and lowest in the month of May-June (5.55±6.69, 1.48±1.90) respectively and this difference is significant statistically (p<0.0001). While for *An*. *fluviatilis*, this trend is different and the highest per trap/night catches of *An*. *fluviatilis* were found in September 1.88±3.95 and nil in the month of June (Fig 3). Comparison of outdoor Vs. indoor catches of LT revealed that more *An*. *culicifacies* and *An*. *fluviatilis* were found in LTO (9.10±15.25, 0.54±2.47) as compared to LTI (8.26±13.33, 0.42±1.30), though not significant (p>0.05).

### Human landing catches (HLC)

**Fig 5:**
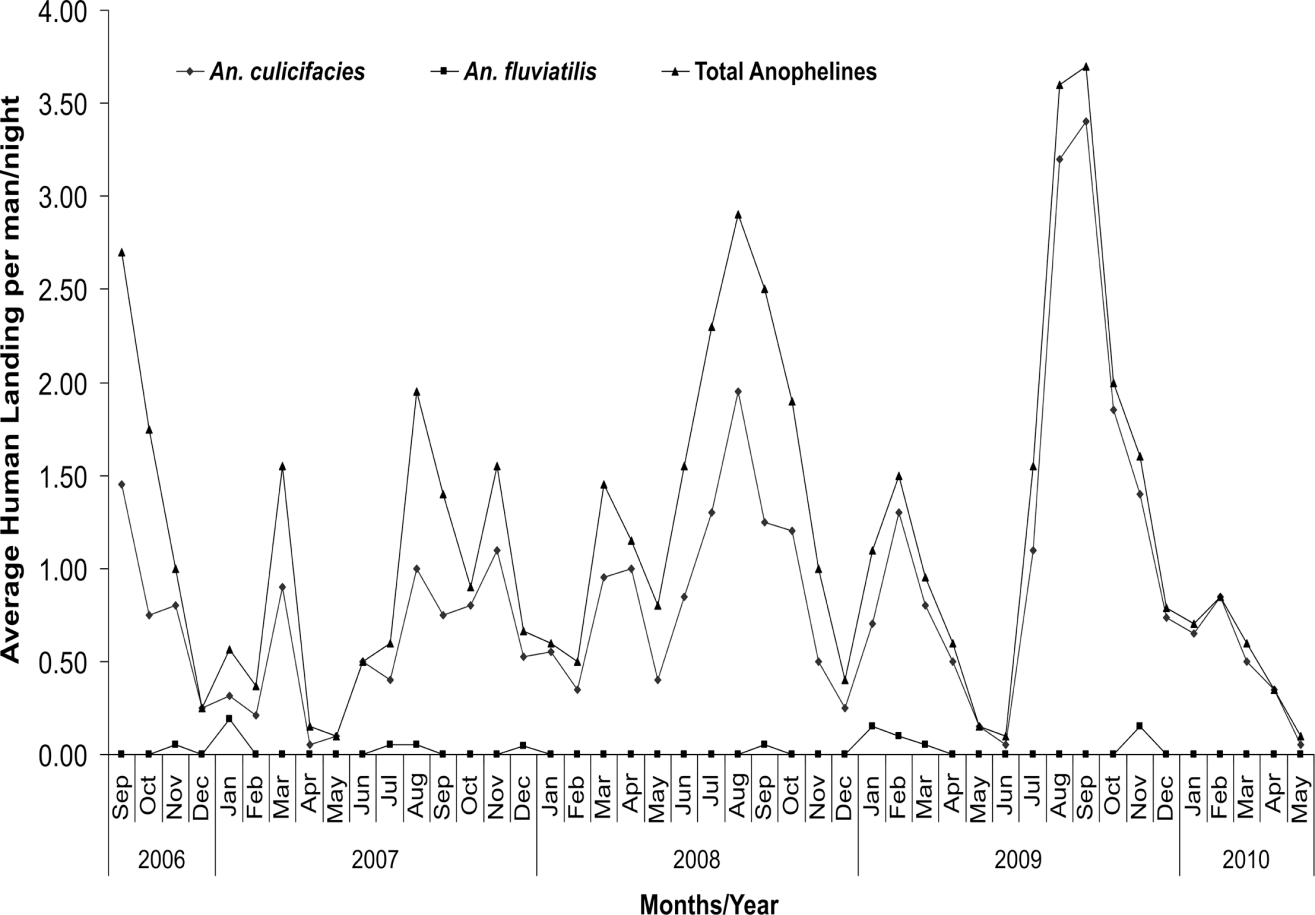
Month/Year wise per man per night catches in study villages of district Jabalpur.

During the course of 221 all night HLC, a total 561 anophelines were captured indoor of which 72% were *An*. *culicifacies*, 1% *An*. *fluviatilis* and 27% were other species. Similarly 498 anophelines were captured outdoor of which 70%, 2% were *An*. *culicifacies* and *An*. *fluviatilis* respectively, while 28% were other species (Table 1). Per bait per night anopheline catches were highest during peak monsoon period i.e. August-September (2.68±3.17) and lowest during peak winter season i.e. December and January (0.50±0.91) (Fig 5). Over the whole period of the study, the catches per man per night were 1.27±1.98 in human landing catches indoor (HLCI) and 1.12±2.05 in human landing catches outdoor (HLCO) respectively for all anopheline species.

*An*. *culicifacies* was the most common species at both sites with 0.92±1.46 per man per night landing indoor and 0.78±1.68 outdoor (p<0.05). *An*. *fluviatilis* landing was relatively less (0.01±0.13 indoors and 0.02±0.15 outdoors). The landing rate of *An*. *culicifacies* in different years of the study (2007–2009) was 0.56±1.02, 0.88±1.45 and 1.27±2.29 respectively and this increasing trend was statistically significant (p = 0.0001). Further analysis revealed that significantly more *An*. *culicifacies* (p<0.001) landed between 1900–2300 hrs than the remaining hours (2400–0600 hrs) (Fig 4).

### Sibling species distribution by ecotypes

Sibling species distribution (cytogenetic and molecular methods) by ecotypes showed (Table 2) that dam village and plain villages were having relatively more *An*. *culicifacies* C (69%) than in forest villages (56%) and foothill villages (51%) and this difference was significant statistically (p = 0.006). Sibling species D was highest in forest villages (30%) followed by foothill villages (28%) and dam village (18%) and lowest in plain villages (14%) and this difference was also significant statistically (p = 0.001). Except these, there was no other major difference between the sites, and pooled data showed that overall out of 583 total *An*. *culicifacies* tested, proportion of species C was highest (59%) followed by D (24%), B (8.7%), E (6.7%) and A (1.5%) [GenBank database accession numbers of *Anopheles culicifacies* A (KP877448—KP877455), *An*. *culicifacies* B (KP877456—KP877463), *An*. *culicifacies* C (KP877464—KP877471), *An*. *culicifacies* D (KP877472—KP877479) and *An*. *culicifacies* E (KP877480—KP877487)].

**Table 2:** Ecotypes and village wise distribution of sibling species and sporozoite positive of *An*. *culicifacies*, *An*. *fluviatilis* in Bargi and Sihora PHCs, Jabalpur, Madhya Pradesh.

Site	Ecotype	Village	*An*. *culicifacies*							*An*. *fluviatilis*			
			A	B	C	D	E	Sibling not tested	Total	S	T	Sibling not tested	Total
			n/d	n/d	n/d	n/d	n/d	n/d	n/d	n/d	n/d	n/d	n/d
Site—A	Dam	Gullapath	0/2	0/8	3/91	0/23	0/7	1195	3/1326	0/1	0/30	2	0/33
	Forest	Bamhni	0/0	0/0	3/14	0/6	0/2	344	3/366	0/0	2/43	19	2/62
		Rengajhori	0/2	0/11	4/73	0/41	0/6	730	4/863	0/0	0/98	27	0/125
	Foothill	Mukanwara	0/2	0/8	3/45	0/22	0/10	542	3/629	0/0	2/74	43	2/117
		Jamuniya	0/0	0/4	3/21	1/12	0/0	411	4/448	0/0	0/24	4	0/28
		Para	0/3	0/11	2/44	0/25	0/9	596	2/688	0/2	0/58	10	0/70
**Total (A)**			**0/9**	**0/42**	**18/288**	**1/129**	**0/34**	**3818**	**19/4320**	**0/3**	**4/327**	**105**	**4/435**
Site—B	Plain	Gauraha	0/0	0/5	0/26	0/2	0/2	106	0/141	0/0	0/2	0	0/2
		Paharua	0/0	0/4	1/30	0/9	0/3	207	1/253	0/0	0/6	1	0/7
		Khalari	0/0	0/0	0/0	0/0	0/0	20	0/20	0/0	0/0	1	0/1
		Hardua	0/0	0/0	0/0	0/0	0/0	35	0/35	0/0	0/0	0	0/0
**Total (B)**			**0/0**	**0/9**	**1/56**	**0/11**	**0/5**	**368**	**1/449**	**0/0**	**0/8**	**2**	**0/10**

n/d—numerator (sporozoite positive)/denominator (number of mosquitoes tested)

Out of 338 total *An*. *fluviatilis* tested for sibling species, most (99%) were species T, only three (one in dam village and two from foothill) were species S (Table 2). For *An*. *fluviatilis* PCR-identified species T and S were further confirmed by DNA sequencing and were found identical to GenBank entry (DQ238490, KC345547, KC345546) corresponding to species T and S respectively.

### Sibling species distribution by sampling techniques

Analysis of *An*. *culicifacies* sibling species revealed that all five species were present at site A by all sampling techniques except that species A was not found in HLCI (Table 3). Sibling species C was relatively more in MHD (64%) and HLCI (64%) as compared to HLCO (48%) and LT (45%). Similarly E was much less in MHD (2.2%) as compared to HLCI (18%) while B was lowest in HLCI (10%) as compared to LTI (31%). The overall proportion of species A was lowest while C was highest by all collection methods at site A. While species A was absent at site B and C and D were the most common species.

**Table 3:** Distribution of sibling species and sporozoite positive of *An*. *culicifacies*, *An*. *fluviatilis* by various sampling techniques in Bargi and Sihora PHCs, Jabalpur, Madhya Pradesh.

Site	Locality/site of collection	*An*. *culicifacies*							*An*. *fluviatilis*			
		A	B	C	D	E	Sibling not tested	Total	S	T	Sibling not tested	Total
		n/d	n/d	n/d	n/d	n/d	n/d	n/d	n/d	n/d	n/d	n/d
Site A	Indoor Resting Collection (MHD)	0/3	0/19	14/173	1/71	0/6	2680	15/2952	0/0	2/227	47	2/274
	Human bait (Indoor)	0/0	0/3	1/25	0/4	0/7	194	1/233	0/0	0/5	0	0/5
	Human bait (Outdoor)	0/2	0/6	0/28	0/14	0/8	150	0/208	0/1	0/8	4	0/13
	Light trap (Indoor)	0/2	0/6	1/33	0/21	0/6	371	1/439	0/1	0/41	25	0/67
	Light trap (Outdoor)	0/2	0/8	2/29	0/19	0/7	423	2/488	0/1	2/46	29	2/76
	**Total (A)**	**0/9**	**0/42**	**18/288**	**1/129**	**0/34**	**3818**	**19/4320**	**0/3**	**4/327**	**105**	**4/435**
Site B	Indoor Resting Collection (MHD)	0/0	0/8	1/43	0/6	0/2	268	1/327	0/0	0/6	1	0/7
	Human bait (Indoor)	0/0	0/0	0/3	0/1	0/0	24	0/28	0/0	0/0	0	0/0
	Human bait (Outdoor)	0/0	0/0	0/3	0/2	0/2	18	0/25	0/0	0/1	0	0/1
	Light trap (Indoor)	0/0	0/1	0/3	0/2	0/1	33	0/40	0/0	0/0	0	0/0
	Light trap (Outdoor)	0/0	0/0	0/4	0/0	0/0	25	0/29	0/0	0/1	1	0/2
	**Total (B)**	**0/0**	**0/9**	**1/56**	**0/11**	**0/5**	**368**	**1/449**	**0/0**	**0/8**	**2**	**0/10**

n/d—numerator (sporozoite positive)/denominator (number of mosquitoes tested)

### Seasonal variations in sibling species

Season-wise analysis revealed that all five sibling species of *An*. *culicifacies* were present in all five seasons at site A (Table 4). Relatively more number of C was found during monsoon than cool autumn. Similarly D and E were relatively less prevalent in monsoon season as compared to cool autumn. While sibling species A was absent in all seasons at site B.

**Table 4:** Season wise distribution of sibling species and sporozoite positive of *An*. *culicifacies*, *An*. *fluviatilis* in Bargi and Sihora PHCs, Jabalpur, Madhya Pradesh.

Site	Season	*An*. *culicifacies*							*An*. *fluviatilis*			
		A	B	C	D	E	Sibling not tested	Total	S	T	Sibling not tested	Total
		n/d	n/d	n/d	n/d	n/d	n/d	n/d	n/d	n/d	n/d	n/d
Site A	Hot Summer	0/3	0/5	7/63	1/26	0/6	723	8/826	0/0	0/6	1	0/7
	Monsoon	0/2	0/9	8/53	0/17	0/4	1094	8/1179	0/0	3/49	37	3/86
	Post Monsoon	0/1	0/5	2/40	0/20	0/8	646	2/720	0/1	1/101	32	1/134
	Cool Autumn	0/1	0/10	0/51	0/32	0/7	607	0/708	0/1	0/88	29	0/118
	Spring	0/2	0/13	1/81	0/34	0/9	743	1/887	0/1	0/83	6	0/90
	**Total (A)**	**0/9**	**0/42**	**18/288**	**1/129**	**0/34**	**3813**	**19/4320**	**0/3**	**4/327**	**105**	**4/435**
Site B	Hot Summer	0/0	0/0	0/9	0/1	0/0	9	0/19	0/0	0/0	0	0/0
	Monsoon	0/0	0/4	1/34	0/6	0/2	308	1/354	0/0	0/0	1	0/1
	Post Monsoon	0/0	0/3	0/4	0/0	0/1	45	0/53	0/0	0/4	1	0/5
	Cool Autumn	0/0	0/0	0/5	0/2	0/2	2	0/11	0/0	0/4	0	0/4
	Spring	0/0	0/2	0/4	0/2	0/0	4	0/12	0/0	0/0	0	0/0
	**Total (B)**	**0/0**	**0/9**	**1/56**	**1/11**	**0/5**	**368**	**1/449**	**0/0**	**0/8**	**2**	**0/10**

n/d—numerator (sporozoite positive)/denominator (number of mosquitoes tested)

### Vector incrimination

Table 5 showed that out of 4769 *An*. *culicifacies* assayed through sporozoite-ELISA, 20 were positive for CS protein of which 12 were reactive for *P*. *falciparum* and remaining for two polymorphs of *P*. *vivax* (five for VK247, one VK210 and two mixed: VK 247 and VK 210). The positive *An*. *culicifacies* were found during most part of the year except in December, January and February. They were caught from human dwelling, cattle sheds and light trap (indoor/outdoor), HLCI and represented sibling species C & D. Only two *An*. *culicifacies* were found positive from LTO (one *P*. *vivax* VK210 and one *P*. *falciparum*). Out of 445 *An*. *fluviatilis* assayed, four were positive for a CS protein of which 2 were reactive for two polymorphs of *P*. *vivax* (one VK 210 and 1 VK 247) and two for *P*. *falciparum*. All four sporozoite positive were sibling species T of which two were collected from LTO, one from CS and one from HD. None of the *An*. *fluviatilis* species S tested were positive for CS protein.

**Table 5:** Month wise results of sporozoite ELISA test.

Month of collection	*An*. *culicifacies* tested	Positive for CSP protein	*An*. *Fluviatilis* tested	Positive for CSP protein
January	338	0	59	0
February	341	0	49	0
March	558	1 (*Pv*247-1)	41	0
April	430	4 (*Pf-*2, *Pv* 247+210–2)	7	0
May	159	2 (*Pv*247)	0	0
June	256	2 (*Pf*)	0	0
July	414	4 (*Pf-*3, *Pv*210-1)	7	0
August	506	1 (*Pf*)	12	0
September	613	4 (*Pf*-2, *Pv*247-2)	68	3 (*Pf*-2, *Pv*210-1)
October	425	0	70	1 (*Pv*247)
November	348	2 (*Pf*)	69	0
December	381	0	63	0
**Total**	**4769**	**20 (*Pf*-12, *Pv*-8)**	**445**	**4 (*Pf-2*, *Pv*-2)**

Ecotype wise analysis revealed that the highest sporozoite rate was observed in *An*. *culicifacies* in forest and foothill villages (0.57% & 0.51% respectively) followed by dam village (0.23%) and lowest in plain villages (0.22%), though not significant (p = 0.494). Similarly, *An*. *fluviatilis* sporozoite rate was highest in forest villages (1.1%) followed by foothill villages (0.9%) and no positive was found in plain villages and village near the dam (Table 2).

## Discussion

Madhya Pradesh is rich in biodiversity and has a tropical climate with high humidity favorable for different vectors, particularly *An*. *culicifacies* and *An*. *fluviatilis* [8], [28]. Studies on the role of sibling species of *An*. *culicifacies* and *An*. *fluviatilis* in MP are limited and therefore this study was undertaken to understand the role of these vectors collected from different sites in malaria transmission. *An*. *culicifacies* is an endophilic species [29] and indoor resting densities of *An*. *culicifacies* remained high throughout the year as recorded earlier from MP [16], [8]. This species is known to maintain malaria transmission from July to October [29], and accordingly, two rounds of IRS (15^th^ June to 31^st^ July and 1^st^ September to 15^th^ October) were recommended under the National Vector Borne Disease Control Programme for interruption of malaria transmission. However, late winter transmission in February and spring transmission in March by *An*. *culicifacies* has already been recorded from undivided MP [30], [31]. In this study, infective *An*. *culicifacies* species C was found in all nine months from March to November. While infective species D was found only in summer. Infective *An*. *fluviatilis* species T was found mainly during monsoon and post monsoon months. *An*. *culicifacies* (C) and *An*. *fluviatilis* (T) are found transmitting both *P*. *vivax* and *P*. *falciparum* in this study. The overall sporozoite rate is 0.42% for *An*. *culicifacies* (0.25% for *P*. *falciparum* and 0.17% for *P*. *vivax*) while for *An*. *fluviatilis* sporozoite rate is 0.9% (0.45% for *P*. *falciparum* and 0.45% for *P*. *vivax*). The reasons for high infectivity among *An*. *culicifacies* and *An*. *fluviatilis* and extended transmission period could be due to the fact that no vector control measures were undertaken during the study period. Moreover, there are innumerous breeding sites and perennial streams which would not only increase the vector density but also increase humidity which in turn might have increased vector longevity.

The analysis further revealed that the MHD of *An*. *culicifacies* and *An*. *fluviatilis* were similar in every year except for differences in seasonal variations that depended on rainfall, temperature and humidity. Results revealed that the mean MHD of total anophelines and *An*. *culicifacies* were relatively much higher during July to September as compared to other months. Similarly most of the *An*. *culicifacies* were trapped during rainy months. HLC revealed that in the cooler month, landing of *An*. *culicifacies* took place mostly in the first half of the night but in the hot months it shifted to second and third quarter of the night and in monsoon, landing was entirely arrhythmic and occurred throughout the night as recorded earlier [16], [32]. In Hazaribagh, Central India, Senior White [33] also found that night prevalence gradually decreased from 2300 to 0500 h supporting the view that the period of highest activity was before 2300 h as observed in this study.

Site wise results revealed that sibling species A of *An*. *culicifacies* and sibling species S of *An*. *fluviatilis* were absent at site B although these sites are not comparable as the terrain is different between the two sites. Habitat wise sporozoite positivity data revealed that most of the sporozoite positives were detected from the mosquitoes which were collected from cattle sheds as the human dwellings are generally very close to the cattle shed or the dwellings are mixed. It is likely that mosquitoes bite in human dwellings and move to cattle sheds for resting as cattle sheds are dark, damp and humid.

Further, there has been an ecological succession of vector species in different areas and there is a need to study the changing pattern of vector behavior as *An*. *culicifacies* which is a known endophilic species is also found positive in outdoor trap catches and thus transmitting malaria outdoor in this study and in an earlier study [8]. Similarly, species S of *An*. *fluviatilis* is known highly efficient vector of malaria in India [3], [12] whereas the role of species T in malaria transmission uncertain. However, in this study species T was found as a vector. This change could be because no current vector control measures were undertaken during the study period or due to some other unknown factors. Moreover, a change in resting preference of *An*. *fluviatilis* (species T) from outdoors to indoors is also recorded. Therefore, further in depth studies are required from other malarious area of the country to confirm this change in behavior. The outdoor abundance of *An*. *culicifacies* (species C) and *An*. *fluviatilis* (species T) was of significance from a malaria control stand point because these vectors may avoid contact with insecticide sprayed inside the houses. These vectors breed in many widely dispersed stream bed pools, slow running streams and seepages, so larval control is especially difficult.

The finding of this study suggests that *An*. *culicifacies species* C is an established vector of malaria in MP in all ecotypes (forest, foothills, near the dam and in plain). While *An*. *fluviatilis* T plays an important role in malaria transmission in forest and foothill areas. The reported variability in malaria transmission of the member species of *An*. *culicifacies* and *An*. *fluviatilis* complexes warrant evaluation of their vector potential in areas of different endemicity. Our study provides crucial information on *An*. *culicifacies* and *An*. *fluviatilis* sibling species distribution in the malaria endemic region of Jabalpur which will assist in developing strategic control measures against *An*. *culicifacies* and *An*. *fluviatilis*.

It is worthwhile to mention here that *An*. *culicifacies* E and *An*. *fluviatilis* T were not found in the earlier study carried out in Jabalpur [3]. *An*. *culicifacies* E is a highly competent vector for malaria globally and in India [3]. Its adaptability has enabled *An*. *culicifacies* E to invade many areas, which were so far free from it. However, in this study we did not find E positive for sporozoite. The high levels of sporozoite-positive *An*. *culicifacies* (species C) found in indoor collections revealed that transmission is taking place inside the house. However, some transmission is taking place outdoors also as infective *An*. *culicifacies* and *An*. *fluviatilis* were found in outdoor catches. In fact, *An*. *fluviatilis* T along with *An*. *culicifacies* C are important in extending the duration of the malaria transmission period well beyond the wetter months. The biggest challenge in malaria control is to reduce the transmission period and the high sporozoite rate. Simple but effective technologies directed against vectors such as long lasting insecticide- impregnated bed nets (LLINs) and complete coverage by the IRS can dramatically reduce malaria transmission [RMRCT Annual report 2013, page 7–10, http://www.rmrct.org/files_rmrc_web/centre%27s_publications/Annual%20report/Annual_Report%202012-13.pdf]. The strength of this study is a monthly collection of mosquitoes from different ecotypes both indoor and outdoor, the use of cytogenetic and molecular techniques for diagnosis of sibling species and their role in species specific malaria transmission. Such a rigorous approach has not been used in vector bionomics studies carried out earlier particularly in MP. The study has several limitations also. Identification of sibling species is done by both cytogenetic studies and DNA sequencing. Cytogenetic studies require half-gravid ovaries which is difficult in primitive field condition. Moreover, PCR assays based on sequence differences within the ITS2 rDNA [25] and 28S-D3 rDNA [24] being able to separate group 1 (A and D) from group 2 (B, C and E) species of *An*. *culicifacies*. More recently, a two-step multiplex PCR assay based on sequence differences within the COII region distinguishes all five sibling species was developed [34]. However, Surendran et al. [35] reported non-usability of COII-PCR to distinguish the species B from species E in Sri Lanka. We could not identify sibling species of all vectors collected by different methods during the study period. Small sample size prevents us to conclude about the role of other sibling species in different seasons. Moreover, the entomological inoculation rate could not be calculated.

Finally we conclude that the prevalence and human landing behaviour of the *An*. *culicifacies* and *An*. *fluviatilis* with higher activity before midnight when people are not sleeping, the higher risk of infection, and outdoor prevalence of *An*. *culicifacies* and *An*. *fluviatilis* pose considerable operational challenge and suggest that an effective intervention to control malaria in this part could be difficult by the introduction of bed nets alone. The simultaneous use of IRS and LLINs might be beneficial. There is a compelling need for longer lasting IRS insecticides as interruption of malaria transmission still relies on vector control measures.
